# A mixed-methods study on the pharmacological management of pain in Australian and Japanese nursing homes

**DOI:** 10.1093/ageing/afae024

**Published:** 2024-02-26

**Authors:** Laura A Dowd, Shota Hamada, Yukari Hattori, Felicity C Veal, Reina Taguchi, Nobuo Sakata, Agathe D Jadczak, Renuka Visvanathan, Eriko Koujiya, Madhu Rajan, Stefan Doube, Ai Suzuki, Maree Bernoth, Helen Rawson, Hiroshi Maruoka, Amelia Wood, Jo Wagner, Dee-Anne Hull, Mizuki Katsuhisa, Justin Turner, Shin J Liau, Emily Reeve, J Simon Bell, Amanda J Cross

**Affiliations:** Centre for Medicine Use and Safety (CMUS), Faculty of Pharmacy and Pharmaceutical Sciences, Monash University, Parkville, Victoria, Australia; Research Department, Institute for Health Economics and Policy, Association for Health Economics Research and Social Insurance and Welfare, Tokyo, Japan; Department of Home Care Medicine, Graduate School of Medicine, The University of Tokyo, Tokyo, Japan; Department of Health Services Research, Institute of Medicine, University of Tsukuba, Tsukuba, Japan; Department of Geriatric Medicine, Graduate School of Medicine, The University of Tokyo, Tokyo, Japan; Unit for Medication Outcomes Research & Education (UMORE), School of Pharmacy and Pharmacology, University of Tasmania, Hobart, Tasmania, Australia; Research Department, Institute for Health Economics and Policy, Association for Health Economics Research and Social Insurance and Welfare, Tokyo, Japan; Department of Health Services Research, Institute of Medicine, University of Tsukuba, Tsukuba, Japan; Heisei Medical Welfare Group Research Institute, Tokyo, Japan; Adelaide Geriatrics Training and Research with Aged Care (GTRAC) Centre, Adelaide Medical School, Faculty of Health and Medical Sciences, University of Adelaide, Adelaide, South Australia, Australia; National Health and Medical Research Council (NHMRC) Centre of Research Excellence in Frailty and Healthy Ageing, Adelaide Medical School, Faculty of Health and Medical Sciences, University of Adelaide, Adelaide, South Australia, Australia; National Health and Medical Research Council (NHMRC) Centre of Research Excellence in Frailty and Healthy Ageing, Adelaide Medical School, Faculty of Health and Medical Sciences, University of Adelaide, Adelaide, South Australia, Australia; Aged and Extended Care Services, The Queen Elizabeth Hospital, Central Adelaide Local Health Network, Adelaide, South Australia, Australia; Division of Health Sciences, Graduate School of Medicine, Osaka University, Osaka, Japan; Royal Australian College of General Practitioners, Victoria, Australia; Aged Care GP, Melbourne, Victoria, Australia; Transform Physio, Hobart, Tasmania, Australia; Department of Health Services Research, Institute of Medicine, University of Tsukuba, Tsukuba, Japan; Charles Sturt University, Wagga Wagga, New South Wales, Australia; Three Rivers Department of Rural Health, Wagga Wagga, New South Wales, Australia; Murrumbidgee Primary Health Network Aged Care Consortium, Wagga Wagga, New South Wales, Australia; Nursing and Midwifery, Monash University, Clayton, Victoria, Australia; Yokohama Aobanosato Geriatric Health Services Facility, Yokohama, Japan; Longridge Aged Care, Naracoorte, South Australia, Australia; Australian Nursing and Midwifery Federation (SA Branch), Adelaide, South Australia, Australia; Southern Cross Care (SA, NT and VIC), Glenside, South Australia, Australia; Division of Health Sciences, Graduate School of Medicine, Osaka University, Osaka, Japan; Centre for Medicine Use and Safety (CMUS), Faculty of Pharmacy and Pharmaceutical Sciences, Monash University, Parkville, Victoria, Australia; Centre de Recherche, Institue Universitaire de Gériatrie de Montréal, Montréal, Québec, Canada; Faculté de Pharmacie, Université Laval, Québec city, Québec, Canada; Centre for Medicine Use and Safety (CMUS), Faculty of Pharmacy and Pharmaceutical Sciences, Monash University, Parkville, Victoria, Australia; National Health and Medical Research Council (NHMRC) Centre of Research Excellence in Frailty and Healthy Ageing, Adelaide Medical School, Faculty of Health and Medical Sciences, University of Adelaide, Adelaide, South Australia, Australia; Centre for Medicine Use and Safety (CMUS), Faculty of Pharmacy and Pharmaceutical Sciences, Monash University, Parkville, Victoria, Australia; Quality Use of Medicines and Pharmacy Research Centre, University of South Australia: Clinical and Health Sciences, Adelaide, South Australia, Australia; Centre for Medicine Use and Safety (CMUS), Faculty of Pharmacy and Pharmaceutical Sciences, Monash University, Parkville, Victoria, Australia; National Health and Medical Research Council (NHMRC) Centre of Research Excellence in Frailty and Healthy Ageing, Adelaide Medical School, Faculty of Health and Medical Sciences, University of Adelaide, Adelaide, South Australia, Australia; Centre for Medicine Use and Safety (CMUS), Faculty of Pharmacy and Pharmaceutical Sciences, Monash University, Parkville, Victoria, Australia

**Keywords:** Pain, Pain management, Nursing homes, Residential aged care homes, Analgesics, Qualitative research, Older people

## Abstract

**Background:**

Understanding how analgesics are used in different countries can inform initiatives to improve the pharmacological management of pain in nursing homes.

**Aims:**

To compare patterns of analgesic use among Australian and Japanese nursing home residents; and explore Australian and Japanese healthcare professionals’ perspectives on analgesic use.

**Methods:**

Part one involved a cross-sectional comparison among residents from 12 nursing homes in South Australia (*N* = 550) in 2019 and four nursing homes in Tokyo (*N* = 333) in 2020. Part two involved three focus groups with Australian and Japanese healthcare professionals (*N* = 16) in 2023. Qualitative data were deductively content analysed using the World Health Organization six-step Guide to Good Prescribing.

**Results:**

Australian and Japanese residents were similar in age (median: 89 vs 87) and sex (female: 73% vs 73%). Overall, 74% of Australian and 11% of Japanese residents used regular oral acetaminophen, non-steroidal anti-inflammatory drugs or opioids. Australian and Japanese healthcare professionals described individualising pain management and the first-line use of acetaminophen. Australian participants described their therapeutic goal was to alleviate pain and reported analgesics were often prescribed on a regular basis. Japanese participants described their therapeutic goal was to minimise impacts of pain on daily activities and reported analgesics were often prescribed for short-term durations, corresponding to episodes of pain. Japanese participants described regulations that limit opioid use for non-cancer pain in nursing homes.

**Conclusion:**

Analgesic use is more prevalent in Australian than Japanese nursing homes. Differences in therapeutic goals, culture, analgesic regulations and treatment durations may contribute to this apparent difference.

## Key Points

- Analgesic use was seven times higher in Australian versus Japanese residents, including 30-fold higher use of opioids.
- Australian and Japanese healthcare professionals described individualised pain management and first-line use of acetaminophen.
- Timing and method of pain assessment, and clinician preferences for analgesia type and duration, differed between countries.
- Different interventions for ensuring safe analgesic use were identified, but may not translate between countries.

## Introduction

Australia and Japan are two counties with rapidly ageing populations [1, 2]. People admitted to Australian and Japanese nursing homes are increasingly older, experience high rates of frailty, cognitive impairment, complex multimorbidity and polypharmacy [3–5]. Effective pain management is important because unrelieved pain has considerable functional, cognitive, emotional and societal consequences [6]. A recent systematic review of international studies found that up to 79% of nursing home residents self-reported experiencing pain, with the prevalence varying according to country, resident mix, method and timing of assessment [7].

Pharmacological and non-pharmacological strategies are often required for optimal pain management [8]. Despite the pivotal role of traditional analgesics (acetaminophen [i.e. paracetamol], non-steroidal anti-inflammatory drugs [NSAIDs] and opioids) as a component of overall pain management strategies, there is substantial geographic heterogeneity in prescribing [9]. Acetaminophen is generally considered the first-line analgesic for mild-to-moderate pain in older adults [8]. Opioid prescribing practice varies between countries and cultures. An Australian-wide study reported that 27% of 203,894 nursing home residents were chronic opioid users in 2016 (95% confidence interval, CI 26.6–26.9%) [10]. In contrast, the 12-month opioid prevalence in Japan’s general population was <0.1% in 2017 [11]. A qualitative study reported primary care physicians from the USA were more likely to believe opioids were used too often in their country, compared with Japanese primary care physicians (95% vs 7%) [12]. Adjuvant medications (gabapentinoids, tricyclic antidepressants [TCAs] and selective serotonin and norepinephrine reuptake inhibitors [SNRIs]) are increasingly utilised to alleviate certain types of neuropathic pain, despite mixed evidence of effectiveness and risks of adverse drug events (ADEs) in older adults [8].

The United Nations and the World Health Organization (WHO) recommend collaboration and knowledge sharing as part of the 2021–2030 ‘Decade of Healthy Ageing’ [2]. Cross-national exchanges of knowledge, perspectives and practice are important to understand potential factors that influence analgesic decision-making and inform initiatives to improve the pharmacological management of pain. Australia and Japan are well placed for cross-national comparisons, given the growing ageing populations, similar resident demographics and models of nursing home care, yet preliminary disparities in analgesic use [3–5, 13]. The objectives of this study were to (1) compare patterns of analgesic use among samples of Australian and Japanese nursing home residents; and (2) explore the perspectives of Australian and Japanese healthcare professionals on analgesic use in nursing homes.

## Method

### Design

This was an explanatory mixed-methods study, consisting of a quantitative component (part one), followed by a qualitative component (part two) [14]. Part one involved a comparison of analgesic use among samples of Australian and Japanese nursing home residents. Part two involved a qualitative exploration of the perspectives of Australian and Japanese healthcare professionals on analgesic use in nursing homes using semi-structured focus groups. Part two was conducted in accordance with the consolidated criteria for reporting qualitative studies statement (Supplementary File S1) [15].

### Setting

This study was conducted in relation to residents in nursing homes. There are parallels between nursing home care in Australia and Japan [13]. Nursing homes in Australia (residential aged care facilities/services) and Japan (special nursing homes or *Tokuyo*) provide supported accommodation for older adults with long-term care needs that can no longer be met in their own homes [16, 17]. In both countries, pain is commonly assessed by nurses, care workers or physiotherapists and analgesics are predominantly charted by off-site general practitioners (GPs) or visiting physicians, dispensed by off-site community pharmacies and administered by nurses or care workers [17, 18]. On-site or visiting pharmacists are involved in ensuring quality use of medicines [18, 19]. Australian residents in our study were representative of the overall nursing home population in Australian with respect to age (median: 89 [interquartile range, IQR 84–92] vs 87 [IQR 84–95]), sex (female: 73% vs 66%) and moderate-to-severe dementia status (59% vs 54%) [20]. Japanese residents in our study were representative of the overall nursing home population in Tokyo with respect to age (median 87 [IQR 83–91] vs mean 86) and sex (female: 73% vs 78%) [21].

### Part 1. Quantitative

#### Sample

For the Australian sample, a secondary cross-sectional analysis of baseline data from the Frailty in Residential Sector over Time (FIRST) study in 2019 was undertaken [22]. In brief, medically stable, permanent residents (living in the homes for at least 8 weeks) were recruited from 12 nursing homes in South Australia. Seven of the 12 homes were located in the metropolitan area, two in the outer metropolitan area and three were regional. Residents considered to be at the end of life (<3 months to live) and those not able to complete baseline assessments in English were excluded.

For the Japanese sample, a secondary cross-sectional analysis of a prospective cohort study in 2020 was undertaken. In brief, residents living in four nursing homes located in Tokyo/Kanagawa prefectures of Japan were invited by nursing home staff to participate in the study. Eleven Australian and 39 Japanese residents were excluded from the sample as they missing demographic, clinical characteristics and/or medication data.

#### Data collection

For the Australian sample, researchers extracted medication data for each resident from scanned medication charts between March–October 2019. Regular medications were defined as those charted in the regular section of the chart and had a regular sequence of administrations. As-needed (i.e. pro re nata, PRN) medications were defined as those charted in the PRN section and were considered as PRN administrations if a nurse had signed and dated the administration time over the preceding 7 days. A full audit of each medication entry was completed.

For the Japanese sample, researchers extracted regular and PRN medication data for each resident from electronic prescription records between October and December 2020. PRN medications in Japan were prescribed for immediate use, making PRN prescriptions equivalent to PRN administrations. The number of residents with PRN administrations over the preceding 7 and 30 days were extracted.

#### Medications

Traditional analgesic and adjuvant medications were categorised using the Anatomical Therapeutic Chemical (ATC) Classification System recommended by the WHO (Supplementary File S2) [23]. Traditional analgesics included acetaminophen (ATC code: N02BE01), NSAIDs (M01A) and opioids (N02A, codeine [R05DA04]). Adjuvant medications included gabapentinoids (N02BF), TCAs (N06AA), SNRIs (duloxetine [N06AX21]) and Neurotropin (a drug used for neuropathic and chronic pain in Japan) [24]. Combination products containing more than one active ingredient (i.e. non-opioid plus opioid combinations [N02AJ]) were considered separate medications. Different formulations and strengths of the same active ingredient were considered one medication. NSAID gels and patches were charted for PRN administration during episodes of pain.

#### Resident characteristics

Demographic characteristics (age, sex) and comorbidities were obtained from resident medical records by trained study nurses. The FRAIL-NH scale, a frailty screening tool specifically developed and validated for nursing homes, was used to assess resident frailty status (score 0–14) [25, 26]. Frailty assessments were undertaken by trained study nurses. Using the FRAIL-NH, residents were categorised as non-frail (0–2), frail (3–6) or most frail (7–14). In the Australian sample, cognitive status was assessed using the 12-item Dementia Severity Rating Scale (DSRS, score 0–54) and were classified as no/minimal impairment (0–11) or cognitive impairment (12–54) [27]. In the Japanese sample, cognitive status was assessed using the Japanese ‘Independence in Daily Living in Older People with Dementia’ scale and were classified as no/minimal impairment (independent, rank I and II) or cognitive impairment (ranks III, IV and M) [28].

#### Analysis

A standardised data collection tool was developed and pilot tested with the investigator team. Analyses were conducted independently in Australia (by LAD) and Japan (by SH). This avoided needing to share resident-level data outside of the respective countries. Descriptive statistics were used to summarise the demographic and clinical characteristics of residents. The point prevalence of regular medications was calculated by dividing the number of residents prescribed the analgesic or adjuvant medication, by the total number of residents in the study samples. The period prevalence of PRN administrations was calculated by dividing the number of Australian residents receiving more than one PRN administration in the preceding 7 days, and Japanese residents receiving more than one PRN administrations in the preceding 7 and 30 days, by the total number of residents in the respective study samples.

### Part 2. Qualitative

#### Participants

Focus group participants were identified via the professional networks of the investigator team. Purposive and snowball sampling was used to recruit a maximum variation, multidisciplinary group of Australian and Japanese healthcare professionals [29]. Eligibility criteria included being a physician, nurse, pharmacist or physiotherapist, with knowledge or experience providing pain management to nursing home residents. The healthcare professionals involved in part two were not involved in analgesic prescribing for residents included in part one. We aimed to recruit six to nine participants for each Australian and Japanese focus group to provide participants with equal opportunities to interact and share their perspectives [30, 31].

#### Data collection

**Figure 1:**
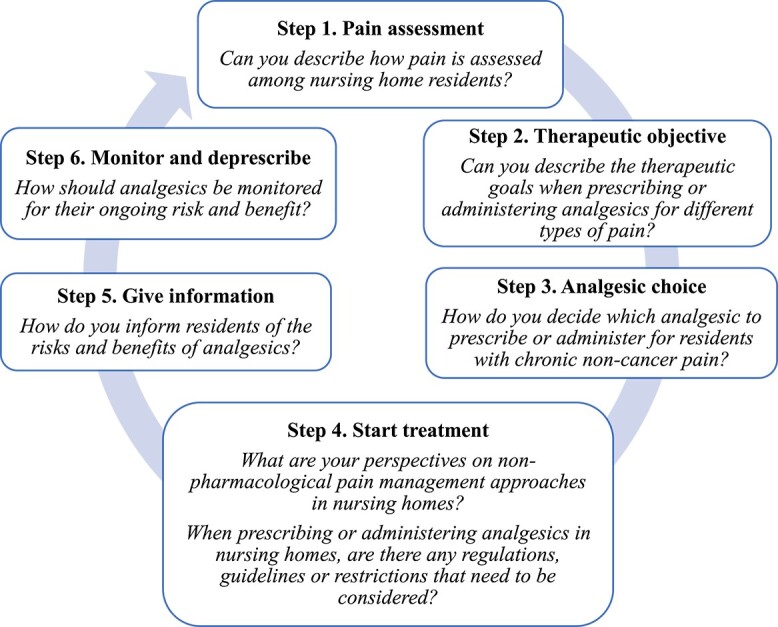
Semi-structured discussion guide, adapted from the WHO six-step Guide to Good Prescribing [32].

Focus groups were conducted using a semi-structured discussion guide developed by the Australian and Japanese investigator team (Australia: LAD, AJC, JSB, Japan: SH, YH, RT) based on the WHO six-step Guide to Good Prescribing (Figure 1, Supplementary File S3) [32]. This framework was consistent with the multidisciplinary contribution towards analgesic use in Australian and Japanese nursing homes. Based on the framework, the discussion guide included seven open-ended questions related to (1) pain assessment; (2) therapeutic goals; (3) analgesic choice; (4) starting analgesic treatment; (5) giving information and (6) monitoring and deprescribing. For steps 3–6, the discussion was focused on chronic non-cancer pain. The semi-structured discussion guide was pilot tested for face validity by two experienced qualitative researchers who were independent from the study.

Three focus groups were conducted in March–April 2023 (Supplementary File S4). Two initial focus groups were held: the Australian focus group was facilitated in English (by LAD) and the Japanese focus group was facilitated in Japanese (by SH). The facilitators and data coders were pharmacists with qualitative research experience and were familiar with medication use in the nursing home setting (Supplementary File S1). Findings from part one were presented to participants at the beginning of both focus groups. The facilitator of the Japanese focus group observed the Australian focus group before facilitating the Japanese focus group. A third cross-national focus group was facilitated in English (by LAD) with three Australian and three Japanese participants. The purpose of the cross-national focus group was to compare, contrast and consolidate the preliminary similarities and differences identified in the country-specific focus groups. Translation assistance was provided by a bilingual investigator (by YH). All focus groups lasted between 60 and 90 min. The focus groups were conducted via a video-conferencing platform (Zoom™), audio-recorded and transcribed. Australian and Japanese transcripts were checked against audio twice by two members of the investigator team (LAD, SH).

#### Analysis

Deductive content analysis was conducted based on the WHO six-step Guide to Good Prescribing [33]. Identifying information was removed from the transcripts prior to analysis. The Japanese transcript was translated verbatim into English and content analysis was performed in English. Data familiarisation and coding of the two country-specific focus groups were conducted independently by two Australian investigators (LAD, AJC), with assistance from a Japanese investigator (SH). These authors met to discuss, compare and refine emergent findings before the cross-national focus group. The preliminary similarities and differences in the country-specific focus groups were presented to the cross-national focus group to clarify and expand on emergent findings. The third focus group was independently coded by the same researchers using the same process as the country-specific focus groups. Themes were finalised through discussion with the entire investigator team. Illustrative quotes from Japanese participants were further validated against the original Japanese transcript by two bilingual members of the investigator team (SH, RT) to ensure the wording and content of the text was accurate. Data were managed using NVivo Pro (Version 20.0, QSR, International Pty Ltd, Australia).

#### Ethics

Part one was approved by the University of Adelaide Human Research Ethics Committee (HREC-2018-247), South Australian Department for Health and Wellbeing Human Research Ethics Committee (HREC/20/SAH/15), Department of Human Services External Request Evaluation Committee (EREC/RMS0432) and was registered with the Monash University Human Research Ethics Committee (23620) and the ethical review board of the Institute for Health Economics and Policy (R2–002) in Japan.

Part two was approved by the Monash University Human Research Ethics Committee (32031) and the ethical review board of the Institute for Health Economics and Policy (R2–002) in Japan. Written informed consent was obtained from all participants prior to the focus groups.

## Results

### Part 1. Quantitative

In total, 550 Australian and 333 Japanese residents were included (Table 1). Australian and Japanese residents were similar in age (median: 89 [IQR 84–92] vs 87 [IQR 83–91]) and sex (female: 73.3% vs 73.3%). A lower proportion of Australian residents were assessed as ‘most frail’ using FRAIL-NH (45.6% vs 61.9%) than Japanese residents. Australian residents had a higher prevalence of regular oral acetaminophen (69.3% vs 3.6%) and opioid (30% vs 0.9%) use, but a lower prevalence of regular oral NSAID (1.5% vs 5.7%) use than Japanese residents. Overall, 74.4% of Australian and 10.5% of Japanese residents used regular oral acetaminophen, NSAIDs or opioids. Australian residents had a higher prevalence of regular adjuvant use and were more likely to be administered a PRN traditional analgesic in the previous 7 days. In the 30 days prior to baseline assessments, 3.3% of Japanese residents used a PRN NSAID patch.

**Table 1:** Resident characteristics, regular and PRN analgesic use in Australia (2019) and Japan (2020)

	**Australia (*N* = 550)**		**Japan (*N* = 333)**	
**Characteristics**				
Age, median (IQR)	89 (84–92)		87 (83–91)	
Female, n (%)	403 (73.3)		244 (73.3)	
Comorbidities, n (%)				
Coronary heart disease	155 (28.2)		39 (11.7)	
Stroke	161 (29.3)		118 (35.4)	
Diabetes	131 (23.8)		74 (22.2)	
FRAIL-NH, n (%)				
Non-Frail (0–2)	68 (12.4)		43 (12.9)	
Frail (3–6)	231 (42)		84 (25.2)	
Most Frail (7–14)	251 (45.6)		206 (61.9)	
Cognitive status, n (%)^a^				
No/minimal impairment	133 (24.2)		102 (30.6)	
Cognitive impairment	417 (75.8)		231 (69.4)	
**Traditional analgesics (regular), n (%)**				
Acetaminophen	381 (69.3)		12 (3.6)	
NSAIDs (oral)^b^	8 (1.5)		19 (5.7)	
Opioids^c^	165 (30)		3 (0.9)	
**Adjuvant medications (regular), n (%)**				
Gabapentinoids	61 (11.1)		13 (3.9)	
TCAs	29 (5.3)		2 (0.6)	
SNRIs	12 (2.2)		5 (1.5)	
Neurotropin^d^	0		5 (1.5)	
**Traditional analgesics (PRN), n (%)** ^**e**^	**Prescribed**	**Admin 7 days**	**Admin 7 days**	**Admin 30 days**
Acetaminophen	299 (54.4)	21 (3.8)	1 (0.2)	3 (0.9)
NSAIDs (oral)	19 (3.5)	0	0	1 (0.3)
NSAIDs (patches)	0	0	1 (0.2)	11 (3.3)
NSAIDs (gel)	14 (2.5)	0	0	6 (1.8)
Opioids	212 (38.5)	38 (6.9)	0	0

IQR, Interquartile Range; NSAIDs, Nonsteroidal Anti-Inflammatory Drugs; PRN, Pro Re Nata (as-needed); SNRIs, Selective Serotonin and Norepinephrine Reuptake Inhibitors; TCAs, Tricyclic Antidepressants

^a^Australian sample: Dementia Severity Rating Scale (DSRS): No/minimal impairment 0–11; Cognitive impairment 12–54. Japanese sample: ‘Independence in Daily Living in Older People with Dementia’ Japanese original scale: No impairment: Independent, Ranks I and II; Cognitive impairment: Ranks III, IV and M.

^b^Oral NSAIDs in the Australian sample were: celecoxib (*n* = 4), meloxicam (*n* = 3) and ibuprofen (*n* = 1). Oral NSAIDs in the Japanese sample were mostly celecoxib (*n* = 16).

^c^Oral opioids used in the Australian sample were: buprenorphine (*n* = 81), oxycodone+naloxone (*n* = 34), oxycodone (*n* = 21), fentanyl (*n* = 21), tramadol (*n* = 11), tapentadol (*n* = 7), acetaminophen+codeine (*n* = 4), morphine (*n* = 2), codeine (*n* = 1) and hydromorphone (*n* = 1). NB: This does not equal *n* = 165 as some residents were prescribed more than one opioid. Oral opioids used in the Japanese sample of residents were Tramadol (*n* = 3).

^d^Neurotropin is a drug used for neuropathic and chronic pain in Japan [24].

^e^PRN traditional analgesics is reported as: number of residents prescribed and administered PRN analgesics in the previous 7 days for the Australian sample and number of residents administered PRN analgesics in the previous 7 days and 30 days for the Japanese sample.

### Part 2. Qualitative

A total of 16 healthcare professionals participated across the three focus groups, including nine in the Australian, seven in the Japanese and six in the cross-national focus group (Supplementary File S4). This included a geriatrician, GP, three pharmacists, three nurses and a physiotherapist in the Australian focus group and two geriatricians/home care physicians, two pharmacists, two nurses and a physiotherapist in the Japanese focus group. The cross-national focus group consisted of a GP, pharmacist and nurse from the initial Australian focus group, and a geriatrician/home care physician, pharmacist and nurse form the initial Japanese focus group. The main findings, including illustrative quotes for the similarities (Table 2) and differences (Table 3) between Australian and Japanese focus groups, are presented for each step of the WHO six-step Guide to Good Prescribing.

**Table 2:** Similarities related to the WHO six-step Guide to Good Prescribing

**Australian healthcare professionals**	**Japanese healthcare professionals**
**Step 1. Identify problem**	
**Importance of family and caregivers for the recognition of pain**	
P8: ‘Quite often we get collateral history from family members, when they notice a change, especially with patients who have cognitive impairment.’	P11: ‘I think it is important for family members or staff to notice changes in the patient’s behaviour, such as changes in facial expressions or a decrease in appetite, which may indicate pain or discomfort.’
**Reluctance of older adults to ‘complain’ about pain**	
P2: ‘The other thing is the reticence of old people to admit that they have pain. Some seem to think that a bit of pain is okay.’	P11: ‘For some other patients, even though they have the same fragility fractures, they do not complain at all and they endure the pain.’ P15: ‘Many older people who have experienced war think that it is great to bear the pain and suffering.’
**Investigating underlying causes of pain**	
P2: ‘Is it psychological pain? Is it physical pain?’	P12: ‘In the case of acute illnesses, there is usually an underlying cause, so it is not appropriate to just watch and wait with analgesics without identifying the cause.’
**Step 2. Therapeutic objective**	
**Individualised approach to pain management, designed to maintain quality of life**	
P9: ‘It is an individualised work-up based on their presentation.’ P5: ‘We want to keep our residents as comfortable as we possibly can, to ensure they have got the quality of life that they are able to live.’	P11: ‘Of course, it depends on the type of pain.’ P15: ‘If the pain is causing problems such as difficulty sleeping or eating, then the goal is to achieve pain relief to the extent that those problems are resolved.’
**Different approaches for cancer-related pain**	
P8: ‘Quite often for cancer pain they already come from the palliative team or the hospital team who have suggested the pain pathway they want us to go down.’ P3: ‘I definitely think there is a lot more conversation with families around the treatment of pain in that end stage.’	P11: ‘In cases where proper and effective control is required, such as with cancer-related pain, it is important to use appropriate medications.’ P10: ‘Unless it is a case where morphine is started in the terminal stage, we usually do not have face-to-face meetings with family members just for the purpose of explaining the medication.’
**Step 3. Treatment choice**	
**Analgesics used in a step-wise approach, with acetaminophen as the first-line analgesic**	
P8: ‘Mostly paracetamol [acetaminophen] is where we start.’	P12 ‘The first step is usually acetaminophen.’
**Concerns of ADEs with adjuvant medications**	
P8 ‘I think gabapentin definitely used to be quite popular. I think I have noticed a change in the last 1 year. All these medications, which have a big anticholinergic drive, they seem to be going out of favour.’	P11: ‘I try to prescribe tramadol or other adjuvant medications, but because of the side effects, as [P8] said, I often have to cease the medication, or try to find alternative medication to ease the pain.”
**Step 4. Start treatment**	
**Non-pharmacological approaches were first-line and should be continued alongside analgesics**	
P9: ‘We are able to offer a number of interventions which include individual prescribed exercises, some receive mindfulness interventions through our OTs [occupational therapists], relaxation, some still receive therapeutic massage if it is appropriate.’	P15: ‘I think that nursing care is the first intervention we take.’ P10: ‘In many cases, we try adding something [non-pharmacological] when dealing with people who regularly use acetaminophen or NSAIDs and still feel some discomfort or pain.’ P13: ‘Some patients prefer a much more natural approach than taking pills. And to think about opioids, almost all patients in Japan do not want to take opioids for non-cancer pain.’
**External regulations influence opioid use**	
P2: ‘They [GPs] were fearful of government reaction to the use of opioids. So, they did not prescribe opioids for older people, even though there was a clear indication for it, because of their fear of getting a government letter.’	P13: ‘For prescribing opioids for chronic pain, prescribing doctors are required to undergo e-learning, and registration is required. Pharmacists who dispense the prescription must confirm that the prescribing doctor has completed the e-learning.’
**Step 5. Give information**	
**Provision of information was more common if resident was perceived to be able to understand and when family was present**	
P6: ‘You engage with family members who are (a) around all the time or (b) shouting the loudest to be perfectly honest with you. Because they are wanting to be informed in every step of the way, so they ask questions.’	P10: ‘It is not very common for family members to visit frequently, so it is quite difficult, or rather unrealistic, to explain every time the medication changes.’
**Pharmacist expertise recognised, but not always involved**	
P5: ‘I think having embedded pharmacists [in nursing homes] coming forward, when the funding finally comes through, there is going to be a massive difference in medication use.’	P16: ‘When patients or family members ask what a certain medication is for, we can provide a simple explanation like “This is a painkiller.” However, we do not have much confidence in explaining the side effects. . . That is why it would be reassuring if pharmacists could explain those things [to patients and their families].’
**Step 6. Monitor, deprescribe**	
**Monitoring pain management through PRN use, ADEs and impact of pain on resident**	
P4: ‘PRN paracetamol [acetaminophen]: we always look at how often they are using that and whether we need to go further.’ P4: ‘On most of them she writes, “Do not hesitate to document when strategies are ineffective, because it is a huge step towards a solution.” And I thought that was just a really good reminder for everyone to document went it does not work, but also document when it does.’	P10: ‘We tend to evaluate patients based on three main aspects: their subjective experience, objective physical findings and behaviours, and the frequency of their use of on-demand [PRN] medication.’ P10: ‘When it comes to NSAIDs, if an older person’s renal function deteriorates rapidly or if they are dehydrated and take NSAIDs, their renal function may rapidly deteriorate. I’m thinking about changing to tramadol or tramadol+acetaminophen to avoid that.’
**Aged care staff and family important in monitoring**	
P1: ‘If the family is actually spending a lot of time visiting them, then at least they are that pair of eyes.’	P15: ‘Ongoing evaluation of how the symptoms are changing and whether they are improving while continuing to use medication is being done by the nurses.’

**Table 3:** Differences related to the WHO six-step Guide to Good Prescribing

**Australian healthcare professionals**	**Japanese healthcare professionals**
**Step 1. Identify problem**	
**Methods of pain assessment**	
**Pain commonly assessed objectively, using validated pain assessment tools** P9: ‘You have your visual analogue scale, your modified verbal inventory of pain. For those with cognitive impairment, the Abbey pain scale would be most common. Occasionally you may see the PAINAD [Pain Assessment in Advanced Dementia] for those with advanced dementia.’	**Pain commonly assessed subjectively, validated tools reserved for cancer pain** P11: ‘I do not use any scales like that [Visual Analogue Scale, Numerical Rating Scale]. Instead, when the patient feels pain, I ask “When do you feel the pain?”’ P10: ‘When it comes to cancer, I feel inclined to use scores or scales, but not so much with regular lower back pain or leg pain. It becomes much more qualitative in those cases.’
**Timing of pain assessment**	
**Routine and periodic pain assessment (e.g. on admission, when there is a change in pain or medications)** P3: ‘Normally on admission, what you would be doing is setting up some pain charting and that generally, depending on the assessment system you have in place, uses validated pain assessment tools. Normally, it is a 7-day pain chart, but also you would do pain charting if you saw a change in pain, or there was a change to medication.’	**Routine pain assessment uncommon, respond to resident episodes of pain as they arise** P11: ‘Basically, our approach is to respond to complaints as they arise.’
**Step 2. Therapeutic objective**	
**Definition of therapeutic goal**	
**Treating until resident is as pain-free as possible** P5: ‘We know if it is [their pain] poorly controlled, they become more sedentary and more at risk of further decline. I guess, what we are looking at is making them as comfortable and pain-free as possible, while allowing them to live the quality of life that they want to or can do.’	**Treating until pain until it no longer interferes with daily life** P15: ‘It is often difficult to achieve zero pain, but the goal is to minimise the impact on daily life, to the point where it does not interfere significantly.’
**Step 3. Treatment choice**	
**Second-line analgesics**	
**Acetaminophen then opioids** P8: ‘I still think the largest use of opioids are oxycodone. Apart from that, opioid patches are pretty popular, especially in patients with dementia.’	**Acetaminophen then NSAIDs then tramadol** P13: ‘There is a huge difference between tramadol to other opioids. Tramadol is treated like “Just a little bit stronger than NSAIDs.” The maximum pain cure for non-cancer pain is tramadol with acetaminophen.’
**NSAIDs rarely prescribed** P8: ‘The only few places where I have prescribed NSAIDs, is when the patients are insisting.’	**NSAID patches popular** P11: ‘Definitely older adults they love NSAID patches, so they seek for NSAID patches.’ P12: ‘We all agree that that the older generations have resistance to pain and oral medications. They handle pain really well. They ask for NSAID patches, but other than NSAID patches, they do not really ask for anything else.’
**Tramadol rarely prescribed** P4: ‘I have seen a lot of adverse effects [with tramadol] and I do not find it overly useful really, especially in older people. And a lot of people are on antidepressants. So, you have got those risks of interactions there.’	**Opioids rarely prescribed** P10: ‘I feel like there is a tendency to avoid using opioids because of these troublesome issues [registration, costs, storage]. It is quite a hassle.’ P12: ‘Due to facility regulations, we are not able to use opioids, so we do not use them very often.’
**Threshold for medication use**	
	**High threshold for medication use** P12: ‘The threshold for whether to use the medication or not is probably a bit high.’ P14: ‘In the beginning, we are quite protective and do not take a very proactive approach.’
**Step 4. Start treatment**	
**Utilisation of non-pharmacological approaches**	
**Analgesic prescribing is often easier than non-pharmacological** P8: ‘Often when you call locums and they do not know the patient, opioids are often the one thing that gets added on.’ P8: ‘In terms on non-pharmacological management, I think this works a bit. But I think all the staff are stressed out. I think the nurses are snowed down with so much work, and it is quite hard for them to cope.’	**Analgesic prescribing often limited as it can be a hurdle** P15: ‘For things like lower back pain and joint pain, it is often a high hurdle to ask for medication, except during regular visits with the contract doctor. In reality, nurses tend to avoid asking [for oral analgesics] and instead try non-pharmacological therapies or nursing care to alleviate the pain, such as using hot compress, massage, and rehabilitation. . .. It is not common for nurses to immediately ask the doctor for oral analgesics for pain caused by musculoskeletal diseases.’
**Step 5. Give information**	
**Step 6. Monitor, deprescribe**	
**Treatment durations**	
**Analgesics often prescribed long-term, deprescribing is a challenge** P4: ‘I would like to see more deprescribing because I do feel it is more of a “Set and forget” at the moment.’ P8: ‘It becomes very hard for us to take them off opioids and reintroduce them back to paracetamol. They see a world of a difference very quickly with opioids and they are quite happy to continue with that.’	**Analgesics prescribed for short durations** P10: ‘If it is a compression fracture and a strong analgesic is prescribed for 1 month, or up to 2 months at the most, acute inflammation at the site is generally resolved. . . Then, I use NSAIDs with the intention of tapering off.” P10: ‘They might say something like, “Take this for 30 days and when you finish it, you can stop taking it” and I think it is sloppy, but in reality, it often works.’
**Facility-level monitoring**	
**Facility-level monitoring** P5: ‘We have an awesome governance committee and we have a regular MAC [medication advisory committee] meetings. We are always looking at medication use and the safety of medicines, qualities of medicines, that sort of thing.’ P1: ‘I just wanted to mention about the Registry of Senior Australians’ [ROSA] monitoring system. I think having a way to benchmark [opioid use] across facilities might actually be a good way to actually try to improve prescribing practices.’	**No facility-level monitoring** P11: ‘But it seems like there is no systematic medication monitoring system.’ P13: ‘There are no special monitoring of this kind [system-level], just ordinarily thing like a regular meeting with all staff, and we discuss about medications and symptoms and everything.’

### Step 1. Identify problem

When asked to describe how pain was assessed among residents, Australian and Japanese participants both discussed the importance of family and caregivers for the recognition of pain. Having this ‘pair of eyes’ on the resident was highly regarded, as both groups mentioned a general reluctance of older adults to express their pain and many residents being unable to communicate their pain. Both participant groups discussed the importance of investigating the underlying causes of pain as part of pain assessment. However, Australian and Japanese participants described different methods and timing of pain assessment. Australian participants described using objective and validated pain assessment tools on admission and when there is a change in pain levels or medication use, which varied based on the nursing home. Japanese participants described using qualitative approaches to general pain assessment, with validated pain assessment tools reserved for cancer-related pain. Japanese participants described responding to episodes of pain as they arise, which were usually escalated by caregivers or rehabilitation staff.

### Step 2. Therapeutic objective

Australian and Japanese participants discussed the importance of an individualised approach to pain management, with their therapeutic goal being to maintain quality of life. However, there were differences in the definition of this therapeutic goal. Australian participants discussed their goal of treating pain until residents were as pain-free as possible. Japanese participants discussed treating pain until it no longer interferes with daily life given the difficulties associated with achieving zero pain. Japanese participants perceived that residents were accepting of a certain level of pain if it did not significantly interfere with daily life.

Both groups discussed how their pain management approach depended on the type and severity of pain. Different approaches for cancer-related pain were discussed compared with non-cancer pain, including greater involvement from healthcare professionals outside the nursing home setting. Japanese participants discussed using objective pain assessment methods and providing comprehensive medication information to residents with cancer-related pain.

### Step 3. Treatment choice

When asked about analgesic selection for residents with chronic non-cancer pain, both groups discussed using analgesics in a step-wise approach. Both groups discussed using acetaminophen as the first-line analgesic, but differed in their choice of second-line analgesics. Australian participants perceived clinicians prescribed opioids for severe pain, with seldom use of NSAIDs or tramadol. Japanese participants reported that NSAID patches were commonly used for Japanese older adults, before or concomitantly with oral analgesics. They perceived that clinicians preferred to prescribe NSAID patches and tramadol for Japanese older adults. They also described older adults often seek NSAID patches when experiencing pain. Japanese participants discussed a judicious approach to oral analgesics, with a combination drug of tramadol and acetaminophen reserved for severe pain, or residents with renal impairment. Australian and Japanese participants perceived that ADEs associated with adjuvant medications (e.g. sedative load and anticholinergic burden) limited the use of these medications. Overall, Japanese participants described a high threshold for the prescribing of oral medications in nursing homes.

### Step 4. Start treatment

All participants considered non-pharmacological approaches were first-line and should be continued alongside analgesics. However, the utilisation of non-pharmacological approaches appeared different between groups. Several Australian participants reported that provision was variable and dependent on the availability of skilled staff to provide non-pharmacological approaches. Japanese participants described the routine, first-line use of non-pharmacological approaches prior to analgesic use. Several Japanese participants perceived that providing non-pharmacological treatment (e.g. hot compress, massage and rehabilitation) was often easier than obtaining an analgesic prescription and that residents usually preferred ‘natural approaches’ to pain management, as opposed to taking oral analgesics.

Australian and Japanese participants described that external regulations influence opioid use, but described different degrees of influence of these regulations. Australian participants reported some GPs were reluctant to prescribe opioids due to fears of an external audit (e.g. receiving a government letter suggesting over-prescribing). Japanese participants reported that prescribers were required to undertake specialised e-learning and registration to prescribe opioids, with pharmacists required to check this registration before dispensing opioids. Japanese participants also described costs (which varied based on the type of nursing home) and strict storage requirements as barriers to using opioids for chronic non-cancer pain in nursing homes. Japanese clinicians discussed that tramadol was often the preferred opioid, because it is not subject to these regulations.

### Step 5. Give information

When asked about the process of providing information to residents regarding the risks and benefits of analgesics, both participant groups reported that provision of information was more common if the resident was perceived to be able to understand and if the family/guardian was present. Australian and Japanese participants recognised the pharmacists’ expertise as a potential provider of information to residents and families, although the extent to which this occurred in routine practice was variable.

### Step 6. Monitor and deprescribe

Australian and Japanese participants discussed the importance of monitoring PRN medication use, ADEs and the impact of pain on residents. Australian participants discussed the value of monitoring opioid use at the nursing home-level, through medication advisory committees and audit and feedback using opioid indicators. There were differences discussed in analgesic treatment durations. Australian participants reported that analgesics are often prescribed for an ongoing and regular basis. They also discussed the difficult task of deprescribing opioids, although were optimistic about improvements in this area. Japanese participants discussed prescribing analgesics for clear, short-term durations that correspond to when a resident reported underlying pain, such as pain due to fragility fracture. Japanese participants appeared to perceive less need for deprescribing interventions.

## Discussion

This was the first study to compare the pharmacological management of pain in Australian and Japanese nursing home residents. Australian residents had a considerably higher prevalence of regular acetaminophen and opioid use, but a lower prevalence of regular oral NSAID use than Japanese residents (part one). There were several similarities and differences in approaches to analgesic use across all domains of the WHO six-step Guide to Good Prescribing (part two), which may contribute to the apparent disparities in analgesic use identified in part one.

Our analgesic comparison identified a 30-fold difference in regular opioid prevalence (30% vs 1%) between samples of Australian and Japanese residents. Opioid prevalence in the Australian sample was consistent with 2022 a systematic review which reported 28–34% of Australian residents used opioids on a regular basis over 1-week to 1-month [34]. Japan’s low opioid prevalence in the general population (<0.1% in 2017) has been previously attributed to cultural contexts, strict regulations, variable understanding of pain and fear of ADEs [11, 12, 35, 36]. In our study, Japanese healthcare professionals reported prescriber training and registration, costs (which varied based on the type of nursing home) and strict storage requirements as barriers to prescribing and storing opioids (excluding tramadol) in nursing homes. These regulations reflect a judicious approach to opioid use in Japan. Most Australian nursing homes have established infrastructure to store and administer opioids [37]. This ease of accessibility may contribute to the overall higher use of opioids in Australia.

Culture and clinician preferences appeared to impact analgesic treatment choice in our study. Japanese healthcare professionals perceived residents had a cultural preference towards non-pharmacological approaches and NSAID patches, attributed to a fear of ADEs. This is consistent with our analgesic comparison, which identified a higher use of NSAIDs, in oral and patch formulation, in the Japanese sample of residents. Conversely, oral NSAIDs were rarely used in the Australian sample, which was consistent with previous literature [38, 39]. Furthermore, NSAID patches are not approved for use in Australia. Australian healthcare professionals perceived clinicians preferred to prescribe acetaminophen and opioids. Opioids are now more prevalent than oral NSAIDs in most countries, due to the risk of ADEs associated with NSAIDs among older adults [9]. Although cognitive impairment was similar between Australian and Japanese samples (76% vs 69%), a lower proportion of Australian residents were assessed as ‘most frail’ using FRAIL-NH (46% vs 62%). Differences in cognitive impairment and frailty may have influenced clinician preferences for analgesic prescribing, although our study did not specifically investigate this. Together, these findings reinforce the benefits of adopting a holistic, multidisciplinary, person-centred approach to pain management, with consideration of resident characteristics, goals of care and preferences throughout [40].

Differences in therapeutic goals and processes for recognising and assessing pain may contribute to differences in analgesic use. Australian healthcare professionals described their therapeutic goal was to alleviate pain and described using validated pain assessment tools on admission and when residents’ clinical circumstances change to ensure the therapeutic goal was achieved. Conversely, Japanese healthcare professionals described their therapeutic goal of minimising the impact of pain on daily activities and perceived residents may be generally accepting of a certain level of pain. Japanese participants described a qualitative approach to pain assessment, which involved responding to episodes of pain as they arise. Japanese older adults are renowned for strong cultural beliefs and silent endurance of pain and discomfort [41]. Previous international studies have suggested that unmanaged or under-managed pain has been largely attributed to difficulties in recognising and assessing pain in older adults, particularly in those with cognitive impairment [42, 43]. In the literature, staff education and implementation of standardised pain assessment tools can improve awareness of pain [44, 45]. Structured approaches to pain assessment that are common in the Australian context may be linked to a greater awareness of pain, hence the higher analgesic use.

Australian healthcare professionals described difficulties deprescribing long-term opioids. This was not raised as an issue by Japanese participants, as they reported prescribing opioids for short-term durations corresponding to acute episodes of severe pain (e.g. pain due to fragility fracture). Specifying treatment durations when initiating analgesics is a known resident-level facilitator for improved prescribing practices, including subsequent deprescribing of analgesics post-resolution of acute pain [40, 46]. Australian healthcare professionals described the value of system-level monitoring of opioid use as a means to improve their safe and effective use. Opioid-related indicators have been proposed as a possible mechanism for nursing homes to monitor safe and effective opioid use [10, 39]. Additionally, chronic opioid use, defined as continuous opioid use for at least 90 days or for 120 non-consecutive days, has been identified as a potential future quality indicator for addition to Australia’s National Aged Care Mandatory Quality Indicator Programme [10]. It is possible that improvements in system-level monitoring of opioid use may impact opioid prescribing practices in Australia.

### Strengths and Limitations

This study utilised an explanatory, mixed-methods approach involving qualitative methods (part two) to explore quantitative findings (part one). In our study, Australian and Japanese residents were similar in terms of age, sex and degree of cognitive impairment. Additionally, the sample of Australian and Japanese residents was broadly representative of nursing home residents in Australia and Tokyo with respect to age, sex and dementia status [20, 21]. The internal validity of the qualitative component was strengthened by having the Japanese facilitator attend the Australian focus group before facilitating the Japanese focus group. Virtual focus groups were chosen to enhance diversity, flexibility and accessibility for participants.

This study was conducted with a relatively small sample of residents (*N* = 883) and healthcare professionals (*N* = 16). The research was explanatory and the findings are not necessarily generalizable to all residents and healthcare professionals in Australia and Japan. Given the cross-sectional nature of our data, we were unable to define analgesic treatment durations. Further research should prioritise exploring cross-national acute vs chronic opioid use, as differences in opioid treatment durations were discussed in the focus groups.

We were unable to compare the prevalence of pain, ethnicities or the provision of non-pharmacological treatments between the Australian and Japanese samples of residents. For this reason, it was not possible to conclude about potential under, over or inappropriate use of analgesics. Additionally, the implications of COVID-19 on analgesic use were not explored in this study. While emerging evidence suggests the prevalence of pain may have increased in nursing homes during COVID-19, changes to analgesic use remains largely unexplored [47]. As with other qualitative research, conceptual interpretation can be dependent on investigator beliefs and experiences [48]. For this reason, coding of qualitative data was performed independently by two investigators, refined with the wider multidisciplinary investigator team and consolidated with the cross-national panel. While we recruited a multidisciplinary group of healthcare professionals, no formal attempts were made to achieve thematic saturation [49].

## Conclusion

Analgesic use is considerably more prevalent in Australian than Japanese nursing homes. Differences in the method and timing of pain assessment, therapeutic goals, culture, clinician preferences, analgesic regulations and treatment durations may contribute to the apparent disparities in analgesic use between the respective countries. Understanding the internal and external influences on analgesic decision-making is crucial for the development of targeted interventions to improve the pharmacological management of pain in nursing homes.

## Supplementary Material

aa-23-1272-File002_afae024
