# Supplementary material for: A mixed-methods study on the pharmacological management of pain in Australian and Japanese nursing homes
Source: Age Ageing. 2024 Feb 26;53(2):afae024. doi: 10.1093/ageing/afae024 (PMC10898334; doi:10.1093/ageing/afae024)
Supplement: aa-23-1272-File002_afae024 [file aa-23-1272-file002_afae024.docx]

**A mixed-methods study on the pharmacological management of pain in Australian and Japanese nursing homes**

**Supplementary File 1.** COnsolidated criteria for REporting Qualitative research (COREQ): 32-item Checklist [15].

**Supplementary File 2**. Anatomical Therapeutic Chemical (ATC) Classification system recommended by the World Health Organization [23].

**Supplementary File 3.** Semi-structured discussion guide, based on the World Health Organization 6-Step Guide to Good Prescribing [32].

**Supplementary File 4.** Flow diagram of qualitative focus groups.

**Supplementary File 1.** COnsolidated criteria for REporting Qualitative research (COREQ): 32-item Checklist [15].

| **No** | **Item** | **Guide questions/description** | |
| --- | --- | --- | --- |
| **Domain 1: Research team and reflexivity** | | | |
| Personal Characteristics | | | |
| 1. | Interviewer/ facilitator | Which author/s conducted the interview or focus group? | LAD facilitated the Australian focus group, with assistance from JSB and AJC. SH facilitated the Japanese focus group, with assistance from YH and RT. |
| 2. | Credentials | What were the researcher's credentials? *E.g. PhD, MD* | At the time of undertaking the study, LADs credentials were BPharm (Hons) and SHs were MSc and DrPH |
| 3. | Occupation | What was their occupation at the time of the study? | LAD is a pharmacist and PhD candidate.  SH is a pharmacist and researcher. |
| 4. | Gender | Was the researcher male or female? | LAD: F  SH: M |
| 5. | Experience and training | What experience or training did the researcher have? | LAD completed an on-line facilitation course (Alison: Facilitation Management). A pilot focus group was held before LAD facilitated the Australian focus group with four senior academics with experience in qualitative research. LAD has previously conducted a survey and facilitated workshops through the University. SH observed the Australian focus group before facilitating the Japanese focus group. |
| Relationship with participants | | | |
| 6. | Relationship established | Was a relationship established prior to study commencement? | Given that participants were recruited through personal contacts, relationships were established prior to study commencement. |
| 7. | Participant knowledge of the interviewer | What did the participants know about the researcher? e*.g. personal goals, reasons for doing the research* | LAD and SH disclosed to all participants that LAD was undertaking this project as part of her PhD. |
| 8. | Interviewer characteristics | What characteristics were reported about the interviewer/facilitator? e.g. *Bias, assumptions, reasons and interests in the research topic* | LAD is a PhD candidate exploring analgesic use in nursing homes, with previous experience as a pharmacist. This information was disclosed to all participants. In order to minimise LAD’s information bias, SH, AJC and JSB were involved in the analysis. |
| **Domain 2: study design** | | | |
| Theoretical framework | | | |
| 9. | Methodological orientation and Theory | What methodological orientation was stated to underpin the study? *e.g. grounded theory, discourse analysis, ethnography, phenomenology, content analysis* | Explanatory mixed-methods design, deductive content analysis. |
| Participant selection | | | |
| 10. | Sampling | How were participants selected? *e.g. purposive, convenience, consecutive, snowball* | Participants were identified via the professional networks of the investigator team. Purposive and snowball sampling was used to recruit a maximum variation group of Australian and Japanese healthcare professionals with different backgrounds. Participants were required to have knowledge and expertise in providing pain management to older adults in nursing homes. |
| 11. | Method of approach | How were participants approached? e*.g. face-to-face, telephone, mail, email* | Participants were emailed and informed about the study by JSB, AJC and SH. If the participants agreed to participate, LAD emailed the explanatory statements and consent forms. |
| 12. | Sample size | How many participants were in the study? | 16 |
| 13. | Non-participation | How many people refused to participate or dropped out? Reasons? | None. |
| Setting | | | |
| 14. | Setting of data collection | Where was the data collected? e*.g. home, clinic, workplace* | Via a video-conferencing platform (Zoom™). |
| 15. | Presence of non-participants | Was anyone else present besides the participants and researchers? | No |
| 16. | Description of sample | What are the important characteristics of the sample? *e.g. demographic data, date* | Clinical background: Geriatricians (2), GP (1), physician (1), pharmacists (4), nurses (5), physiotherapist (2). |
| Data collection | | | |
| 17. | Interview guide | Were questions, prompts, guides provided by the authors? Was it pilot tested? | Focus groups were conducted using a semi-structured discussion guide based on the WHO 6-step Guide to Good Prescribing (**Figure 1, Supplementary File 3**). This was adapted to include seven open-ended questions related to; (1) pain assessment; (2) therapeutic goals; (3) analgesic choice; (4) starting analgesic treatment; (5) giving information and (6) monitoring and deprescribing. The semi-structured discussion guide was pilot tested for face validity by two experienced qualitative researchers who were independent from the study. |
| 18. | Repeat interviews | Were repeat interviews carried out? If yes, how many? | The cross-national focus group, including three participants from each of the country-specific focus groups took place three weeks after the country-specific focus groups. The purpose of this focus group was to compare, contrast and consolidate the preliminary similarities and differences identified in the country-specific focus groups. |
| 19. | Audio/visual recording | Did the research use audio or visual recording to collect the data? | All focus groups with audio and video recorded. |
| 20. | Field notes | Were field notes made during and/or after the interview or focus group? | No. |
| 21. | Duration | What was the duration of the interviews or focus group? | All focus groups lasted between 60 and 90-minutes. |
| 22. | Data saturation | Was data saturation discussed? | No formal attempts were made to achieve data saturation (see Implications, Strengths and Limitations). |
| 23. | Transcripts returned | Were transcripts returned to participants for comment and/or correction? | No. |
| **Domain 3: analysis and findings** | | | |
| Data analysis | | | |
| 24. | Number of data coders | How many data coders coded the data? | LAD and AJC, with assistance from SH. |
| 25. | Description of the coding tree | Did authors provide a description of the coding tree? | Deductive content analysis was conducted based on the WHO 6-step Guide to Good Prescribing. |
| 26. | Derivation of themes | Were themes identified in advance or derived from the data? | In advance. |
| 27. | Software | What software, if applicable, was used to manage the data? | NVivo software release 1.3 (535). |
| 28. | Participant checking | Did participants provide feedback on the findings? | Yes, the purpose of the cross-national focus group was to clarify and expand on emergent findings. |
| Reporting | | | |
| 29. | Quotations presented | Were participant quotations presented to illustrate the themes / findings? Was each quotation identified? e*.g. participant number* | Yes (Table 2 and Table 3). Participant number (P1-P16) are provided for illustrative quotes. |
| 30. | Data and findings consistent | Was there consistency between the data presented and the findings? | Illustrative quotes are presented in a manner consistent with the findings. |
| 31. | Clarity of major themes | Were major themes clearly presented in the findings? | Yes. Results are presented for each step of the WHO 6-step Guide to Good Prescribing, with illustrative quotes in Tables 2 and 3. |
| 32. | Clarity of minor themes | Is there a description of diverse cases or discussion of minor themes? | Yes, within each step of the WHO 6-Step Guide to Good Prescribing. |

**Supplementary File 2**. Anatomical Therapeutic Chemical (ATC) Classification system recommended by the World Health Organization [23].

| **Traditional analgesics** | | | **Specific ATC codes** |
| --- | --- | --- | --- |
| Salicylic acid derivatives (**N02BE01**) | Acetaminophen (i.e. paracetamol) | Acetaminophen (i.e. paracetamol) | N02BE01 |
| Anti-inflammatory and antirheumatic products, non-steroids (**M01A**) | NSAIDs | Ibuprofen | M01AE01 |
|  |  | Diclofenac | M01AB05 |
|  |  | Meloxicam | M01AC06 |
|  |  | Naproxen | M01AE02 |
|  |  | Mefenamic acid | M01AG01 |
|  |  | **COX-2 inhibitors** | |
|  |  | Celecoxib | M01AH01 |
| Opioids in combination with non-opioid analgesics (**N02AJ**) | Acetaminophen and opioid combinations | Acetaminophen + Codeine | N02AJ06 |
|  |  | Acetaminophen + Tramadol | N02AJ13 |
| Opioids (**N02A**), including codeine (**R05DA04**) | Opioids | **Weak** | |
|  |  | Codeine | R05DA04 |
|  |  | Tapentadol | N02AX06 |
|  |  | Tramadol | N02AX02 |
|  |  | **Strong** | |
|  |  | Buprenorphine | N02AE01 |
|  |  | Fentanyl | N02AB03 |
|  |  | Hydromorphone | N02AA03 |
|  |  | Morphine | N02AA01 |
|  |  | Oxycodone | N02AA05 |
|  |  | Oxycodone + Naloxone | N02AA55 |
| **Adjuvant medications** | | |  |
| Gabapentinoids (**N02BF**) | Gabapentinoids | Gabapentin | N02BF01 |
|  |  | Pregabalin | N02BF02 |
| Non-selective monoamine reuptake inhibitors (**N06AA**) | TCAs | Amitriptyline | N06AA09 |
|  |  | Nortriptyline | N06AA10 |
|  |  | Dosulepin/dothiepin | N06AA16 |
|  |  | Imipramine | N06AA02 |
| Other antidepressants (**N06AX**) | SNRIs | Duloxetine | N06AX21 |
| Other | | Neurotropin | N/A |

**Supplementary File 3.** Semi-structured discussion guide, based on the World Health Organization 6-Step Guide to Good Prescribing [32].

Thank you for agreeing to participate in this study. The aim of this study is to explore the perspectives of Australian and Japanese healthcare professionals on the pharmacological management of pain among nursing home residents.

For the purpose of this study;

- The “*pharmacological management of pain*” is the process of alleviating pain using analgesic and/or adjuvant medications.
- *“Analgesic medications”* include paracetamol, nonsteroidal anti-inflammatory drugs [NSAIDs] and opioids.
- *“Adjuvant medications*” include medications that are typically used for indications other than pain management, that provide control of certain pain types (gabapentinoids, tricyclic antidepressants [TCAs] and selective serotonin and norepinephrine reuptake inhibitors [SNRIs]).
- “*Nursing homes*” are services which provide supported accommodation for older adults with long-term care needs that can no longer be met in their own homes.

Please introduce your name, professional background and your experience with providing pain management to older adults living in nursing homes.

Step 1. Identify problem

**Can you describe how pain is assessed among nursing home residents?**

Prompts:

- How involved are you in the assessment of pain?
- What do you think about self-report vs observational pain assessment tools?
- How often should pain assessment should be performed in nursing homes?

Step 2. Therapeutic objective

**Can you describe the therapeutic goals when prescribing or administering analgesics for different types of pain?**

Prompts:

- Different types of pain include acute, chronic non-cancer pain, chronic cancer-related pain/end-of-life pain, nociceptive vs neuropathic, fragility fractures due to osteoporosis
- Is the goal always zero pain? Other goals may include improved quality of life, minimized adverse drug events (ADEs), maximized mobility

*For the following questions, the focus will be on the management of chronic non-cancer pain (pain lasting beyond the time of tissue healing or for over three months) in people living in nursing homes.*

Step 3. Treatment choice

**How do you decide which analgesic to prescribe or administer for residents with chronic non-cancer pain?**

Prompts:

- What are your perspectives on traditional analgesics? E.g. nonsteroidal anti-inflammatory drugs [NSAIDs], paracetamol and opioids
- What are your perspectives on adjuvant medications? E.g. gabapentinoids, tricyclic antidepressants [TCAs] and selective serotonin and norepinephrine reuptake inhibitors [SNRIs]
- Which resident characteristics may influence your analgesic choice? E.g. frailty, cognitive impairment, type of painful condition
- When would you prescribe a PRN analgesic?
- For residents prescribed a PRN analgesic, when would you administer the analgesic?

Step 4. Start treatment

**What are your perspectives on non-pharmacological pain management approaches in nursing homes?**

Prompts:

- Are these approaches used in your practice? Why/why not?
- Which non-pharmacological approaches do you consider most effective? E.g. massage, tai chi
- Should these approaches be used before starting analgesics, alongside analgesics, or both?

**When prescribing or administering analgesics in nursing homes, are there any regulations, guidelines or restrictions that need to be considered?**

Prompts:

- Australian examples: Pharmaceutical Benefits Scheme (PBS), medication reviews
- Japanese examples: Universal long-term care insurance (LTCI) system

Step 5. Give information/instruction

**How do you inform residents of the risks and benefits of analgesics?**

Prompts:

- If you do not, whose role is it?
- Do you involve the family or guardian of residents?
- How do you ensure residents understand the risks associated with their analgesics? E.g. for people living with dementia, poor health literacy, deaf or hard of hearing
- Would you like to do this? What limits your ability to do this?

Step 6. Monitor response

**How should analgesics be monitored for their ongoing risk and benefit?**

Prompts:

- Which staff should be involved in monitoring the effectiveness of analgesics? E.g. nurses, clinical pharmacists, care workers
- What are your main concerns for residents continuing on opioids long-term?
- When do you think analgesics should be stopped/trialled for stopping?
- Are you aware of any facility-level monitoring?
  - Australian examples: Medication Advisory Committees (MACs), medication indicators for bench-marking
  - Japanese examples: No standard guidelines in Japan, *frequent monitoring by care workers for side-effect such as dizziness, vertigo*

Any other thoughts/comments

**Supplementary File 4.** Flow diagram of qualitative focus groups.

Japanese focus group:

- Geriatrician/home care physician (2), pharmacists (2), nurses (2), physiotherapist (1)
- Introduction
- Semi-structured discussion guide
- Analgesic findings from part one

Australian focus group:

- Geriatrician (1), GP (1), pharmacists (3), nurses (3), physiotherapist (1)
- Introduction
- Semi-structured discussion guide
- Analgesic findings from part one

Cross-national focus group:

- Australia: GP (1), pharmacist (1), nurse (1)
- Japan: Geriatrician/home care physician (1), pharmacist (1), nurse (1)
- Introduction
- Analgesic findings from part one
- Emergent similarities/differences from the country-specific focus groups presented
- Compare, contrast and consolidate themes
